# Child Life under Queen Victoria

**Published:** 1897-05-01

**Authors:** 


### Child Life under Queen Victoria.

### XII.—What Remains to be Done.

It does not follow that because much has been done to improve the condition and promote the welfare of children during the last sixty years we have reached an ideal state, and can sit down contentedly with the conviction that all has been done that legislation and philanthropy can do. There are still, in this our England, unhappy children, children who are overworked, maltreated, neglected. But for the most part these cannot be legislated for directly, and even in suggesting remedies we are trespassing beyond the limits to which our title professes to confine us.

Factory legislation was begun in the interests of children, but in its progress it came to embrace those of women also. Personally, as women workers, female factory hands are fairly well protected, but as mothers, actual or potential, their present state cannot be regarded as satisfactory. To forbid the employment of married women in factories might seem an arbitrary act, and it cannot be denied that it might produce evils of its own; at the same time it is so obvious that the proper place for the matron, and above all the mother, is the home, that one might risk something to enfore a command which nature so distinctly enunciates. The present law forbids the employment of women in a factory within four weeks after child-birth. Now, though four weeks' rest may be enough for the mother, four weeks' care is certainly not enough for the child. To abandon a month-old child to such care as the poor can afford to hire, is to give it as bad a chance of long and healthy life as well may be. The baby is entrusted perhaps to the charge of some slightly elder sister, or youthful aunt, not yet of working age. Or some neighbour, whose own progeny are so numerous as to make it impossible for her to go out to work, allows this extra child to be left in her house while its mother is busy. Or some old woman, past work herself, earns a scanty living by keeping a sort of crèche. In the first case the hapless infant has an extra chance of being deformed by careless holding, of catching a fatal chill while the child-nurse gossips with a friend in the street, and of being run over by a passing vehicle. But in all it is equally likely to suffer from unsuitable food, from dirt, and from neglect. If these things make it fretful it is drugged. Doubtless, some children survive this treatment, though with health impaired for life; but to hear mothers of that class dwell, not without a Dogberry-like complacency, on the number they have "buried"—they do not say killed, though perhaps that were the more accurate word—would convince the listener that the children's life is often sacrificed to the mothers' wages.

Men and women are the wealth of a country; children are its investments, and will not produce a good return unless properly cared for. From this point of view, the work of married women outside their own homes is a thing always to be deprecated. We do not here touch on economic objections to the labour of married women; but, looking at it entirely from the standpoint of the welfare of their children, we protest against it as unwise.

Another victim of industrialism is the "half-timer." The professed intention of the law which permits the existence of the half-timer is to permit of parents in necessitous circumstances eking out a subsistence by the help of their children. "Necessitous circumstances" is a relative term. Ask a millionaire if he needs a few shillings more a week, and it is likely enough that you will receive an affirmative answer. To judge by the number of half-timers at the schools in every district where child-labour can be employed, all the parents in the place are necessitous. The half-timer is, as a matter of fact, a concession to the old false sentiment which justified children being set to maintain themselves and help their parents as soon as possible. Of its probable effect on the wages of those parents we do not speak here; but it obviously does away with half of the benefits which the Factory Acts and the Education Acts are designed to confer. By an order issued in 1892 the lowest age at which a child can be employed in a factory is eleven; from eleven to thirteen he may work for half-time. Nominally, therefore, his two years' education is reduced to one; as a matter of fact his schooling becomes a heavy burden to him, and little profit. The half-timer either attends school one half of the day and works at the factory during the other half, or he goes to school one day and to the factory the next. The latter plan is perhaps less bad than the former; the child comes fresh in the morning and has the whole day for study; whereas under the other system the teacher gets his pupil only when exhausted in mind and tired in body. But the desultoriness of the study is in either case apt to be fatal to progress, all the more that it is in the higher standards, which children of that age are entering, that a call begins to be made on the personal intellligence of the pupils, and the mechanism of the three R's ground in in the lower standards is applied to wider fields. As an introduction to individual thought and culture these years are as important to a child's education as any that go before. Without them, indeed, all that has gone before often leaves little permanent impression, and it is much to be regretted that a child should be deprived of his best chances of development for the sake of a few shillings a week. Physically, also, the half-timer suffers, and all who come in contact with these children declare that they look old, tired, and stunted.

Special consideration needs also to be given to pauper children, who are, in a special way, the wards of the State. Where it is at all possible children should not be kept in a workhouse, nor even in a home with other pauper children and the inevitable routine of an institution. Such children when sent out into the world are unfit to hold their own in it, and either fail in the strife or drift into evil ways. It is better to board out pauper children in families of the working-class, where they learn the rough and the smooth, the give and take of life, and are, from the beginning, a part of that world in which they must pass their lives. Of course, some personal care must be taken, some personal supervision exercised, to see that the little pauper boarded is not bullied or ill-treated; but this is by no means unattainable, and in nine cases out of ten it will be found that a child brought up in an ordinary home is brighter, happier, and more intelligent than one whose youth has been passed in the shadow of the workhouse. Some such steps as these would well complete the work that has been done for children during Her Majesty's reign.

